# Analyzing the Relationship between Supervisors and Post-Graduates Based on Differential Game Theory

**DOI:** 10.3390/bs13050414

**Published:** 2023-05-15

**Authors:** Fangfang Liu, Ning Fan, Jiangjun Zhu, Chaoping Li, Shaobo Xie

**Affiliations:** 1 School of Marxism, Chang’an University, Xi’an 710064, China; 2 School of Automobile, Chang’an University, Xi’an 710064, China; 3 School of Public Administration and Policy, Renmin University, Beijing 100872, China

**Keywords:** supervisor–postgraduate relationship, system dynamics, differential game, benefit

## Abstract

A healthy relationship between supervisors and postgraduates is critical for their academic achievements and personal development. This paper quantitatively discusses such a relationship from the viewpoint of differential game theory. First, a mathematic model was established to describe the evolutionary dynamics of the academic level of the supervisor-postgraduate community, which is related to the two parties’ positive and negative efforts. Then, the objective function aimed at maximizing the individual and total benefit of the community was constructed. After that, the differential game relationships in the non-cooperative, cooperative and Stackelberg scenarios were formulated and solved. A comparison of the three game scenarios showed that the optimal academic level and total benefit of the community were 22% higher in the cooperative scenario than in the non-cooperative and Stackelberg game scenarios. Moreover, the influence of model parameters on the game results was analyzed. The results indicate that, for the supervisor-led Stackelberg game, when the sharing cost ratio is increased to a specific level, the supervisor’s optimal benefit will not be further improved.

## 1. Introduction

### 1.1. Background

In recent decades, with the rapid development of the higher education system and increasing employment pressure, more and more undergraduate students chose to pursue postgraduate degrees. As of the end of 2021, the population of postgraduates in China alone had risen to more than 313 million [1]. In this context, exploring the supervisor–postgraduate relationship (SPR) became imperative, as this relationship highly influences the postgraduate’s cultivation quality and academic achievements [2,3], which are essential indicators of evaluating for research-oriented universities [4].

Teachers and students are mainly linked by generalized education activities [5]. The relationship between teachers and students in primary and middle schools is mostly developed in the teaching and learning processes. As the self-consciousness of young students has not yet awakened, and the things they are expected to learn are relatively easy, the relationship between teachers and students at this stage is simple [6]. However, the connection between teachers and university students is more intense. In particular, the interactions between supervisors and postgraduate students become more frequent and deeper [7]. This is due to the students’ improved self-consciousness, their ability to think and decide independently, as well as the higher academic requirements, which require more guidance and mentoring from the supervisors [8].

In reality, the complicated relationship between a supervisor and a postgraduate student lies in many aspects. Firstly, both parties have many common goals. They both desire to improve their academic levels and to gain academic achievements and good reputations. For that, they usually collaborate to conduct research and publish articles. In these situations, their efforts are complementary, and their respective advantages come to the fore. The supervisor’s experience and insights can help the postgraduate student seek proper research topics, while the postgraduate student’s vitality and creativity generate innovative ideas in the scientific works. Nonetheless, due to different cognitions of the SPR, ideological make-up and interest demands, disagreements frequently occur between supervisors and postgraduates [9,10,11]. They may make different decisions, or even completely opposing decisions, in their academic activities. In China, a typical example of this conflict between the supervisor and postgraduate would be deciding on the scientific research condition. Specifically, the supervisor expects the postgraduate student to harvest more academic achievements based on the existing research conditions (since better research conditions mean higher investment and supervisor time costs). However, postgraduate students always complain about the limited and outdated conditions available for scientific research. Another frequent disagreement is about the research topic. Most postgraduate students tendentiously choose either easy research topics (in order to meet the minimum academic requirements), or application-oriented topics, which are conductive to seeking jobs. The supervisors, however, expect the students to take on more challenging topics, in order to make more academic contributions. Therefore, these disagreements can lead to a non-cooperative scenario between the supervisors and postgraduate students. Unlike the cooperative scenario where the supervisor and postgraduate both aim to maximize the interest of the community, in the non-cooperative scenario, both always maximize their individual interests, while neglecting the total interest of the community.

Moreover, as highly socialized individuals, apart from heavy academic missions, many postgraduate students are also subject to non-academic pressures, such as economic pressure [12]. Some have to take part time work to earn enough for tuition and living expenses, and some even assume debts [13]. The conflict between their social position and economic condition could lead to frustration, self-doubt and the students making a negative evaluation of themselves. Reportedly, more than 30% of postgraduate students face high academic and psychological pressure [14]. At the same time, the supervisors, to some extent, nowadays face the academic pressures associated with their universities’ increasing academic requirements. This is especially true of young supervisors, who have not yet obtained the tenure-track [15]. These external pressures on both supervisors and postgraduate students are liable to cause disharmony and ultimately a non-cooperative relationship between them.

Furthermore, the phenomenon of abusive supervision occurs now and then [16]. Currently, the supervisor-responsible system is prevalent across the world in postgraduate education [17]. In such a supervisor-led system, the supervisors have the power to determine whether or not the postgraduate students meet the academic requirements. This results in numerous instances of disharmony. In China, some examples of disharmony are as follows: aiming for private profits, some supervisors require postgraduate students to conduct scientific projects that are completely unrelated to the postgraduates’ research topics [18]. Some supervisors even threaten postgraduate students with delaying their graduation date [19]. The dissatisfaction and complaints from the postgraduate students then transform to inefficient devotion and negative behaviors with regard to the academic activities. The postgraduate students will express this negative emotion, for example, by anonymously conveying their discontentment through a social network, leading to the supervisor getting a bad reputation. Then, these supervisors have to improve their attitudes and behaviors as feedback to the criticism. That is to say, the supervisor’s behavior firstly influences the postgraduate students, and the latter’s behavior will conversely influence the former’s decisions. This is precisely how the supervisor-led game scenario between the two parties is formed.

Summarily, from the viewpoint of game theory, the relationship between a supervisor and postgraduate may occur in different game scenarios, including the cooperative, non-cooperative and supervisor-led scenarios.

### 1.2. Literature Review

The SPR topic was extensively focused upon, and the related literatures can be classified into the two categories of qualitative and quantitative. Both the role and the function of the SPR in a successful postgraduate program were emphasized [20,21]. A healthy and sustainable SPR can improve the overall satisfaction with postgraduate education and the quality of theses [22,23]. An excellent supervisor can provide not only expertise, mentoring and experience [24,25], but also provide encouragement and spiritual comfort to postgraduate students, all of which is beneficial to their academic achievements [26]. Additionally, the SPR is developed in many forms, such as the master–apprentice style [27], the scientific research partners style [28] and the manager-employee style [16]. Moreover, several critical factors related to the SPR were especially closely studied, such as the effect of a postgraduate’s gratitude. Howells et al. [29] studied the interrelation between gratitude and an enhanced relationship between the supervisor and doctoral research students. The authors argued that gratitude can promote communication, social and emotional well-being, and even the research itself. Unsworth et al. [30] discussed the grateful affect and expression within low- and high-trust SPRs. The results showed that the perceptions of supervisors’ altruism and the perceived value of supervisors’ behaviors were positively related to the grateful affect felt by postgraduates in low-trust working relationships. In terms of the quantitative research about the SPR, the conclusions were mostly based on statistical data and information acquired through questionnaires. Using longitudinal data, Liang et al. [31] evaluated the influence of SPR on postgraduate students’ subjective well-being in China. The authors divided SPR into two dimensions, namely the structural and affiliation dimensions. Mainhard et al. [32] and Ma et al. [33] designed questionnaires to investigate SPR from the aspects of influence and proximity. Meanwhile, several theories, such as the cognitive evaluation theory, social exchange theory [34] and informal organization theory [31] were applied to the study of SPR.

Game theory, as an advanced method used to analyze the interactive relationship between two players, was broadly applied in many fields, including economics [35], social behavior [34], management science [36] and STEM fields [37]. The use of game theory to explore the relationship between teachers and students was already documented. Correa et al. [38] discussed the student–teacher relationship by employing a game theory and economic approach, where the Cobb–Douglas production function and the consumer preferences are referred to in the modeling. The authors addressed the conditions and existence of a non-cooperative equilibrium, and pointed out that a non-cooperative equilibrium will result in insufficient academic effort. Correa [39] further discussed the interaction between one teacher and several students by considering their different abilities and attitudes toward work. Gong et al. [40] proposed using the Stackelberg-based game to obtain the equilibrium and incentives involved in the collaboration of supervisors and postgraduates in writing articles. The authors suggested that the university should stimulate the postgraduate students and supervisors with different strengths. Du et al. [41] interpreted the forming mechanism of the game relationship between a supervisor and postgraduate students using the evolutionary game theory.

### 1.3. Aim of Present Study

The gap in existing research lies in two aspects. First, although the supervisor-postgraduate relationship already received considerable attention, the existing studies mainly provide elaborations and analyses from different viewpoints, such as the sociological, valuable and ethnical viewpoints [42,43]. These studies typically employed questionnaire data to assist them in reaching their conclusions. Despite the fact that game theory is involved, few literatures established a system dynamic model of the relationship based on the differential equation; nor did they discuss their differential game relationships. As such, quantitative conclusions cannot be well obtained. Moreover, there are different game scenarios between supervisors and postgraduate students, such as the cooperative, non-cooperative and Stackelberg game models. Therefore, comparing these game results is highly necessary. To fill the current research gap, this paper constructs a mathematical model from the perspective of system dynamics and further discusses the relationship between supervisors and postgraduate students using differential game theory.

This paper proposes an essential framework to discuss the supervisor–postgraduate relationship. This is achieved by establishing a differential model and using differential game theory. Moreover, this methodology can be extended to other interpersonal relationships. The contribution of this paper lies in three aspects. First, the mathematical model used to describe the relationship between the supervisor and postgraduate student is constructed from the perspective of system dynamics. This model considers the academic level of the supervisor–postgraduate community as the state variable, while the positive and negative efforts are the control variables. Thereby, the model is capable of avoiding having the complicated model characteristic that involves many factors. Second, based on the established differential dynamic model, the objective function for maximizing the individual benefits of the supervisor and postgraduate, and the total benefit of both can be formulated. In addition, the differential game relationship is formulated in three typical scenarios, including the cooperative, non-cooperative and Stackelberg mode. The equilibrium strategies are solved and compared for the three modes. Moreover, the results are illustrated, and the sensitivity of the model parameters is further discussed.

The rest of the paper proceeds as follows: in Section 2, the dynamic model of the supervisor–postgraduate relationship is established, and the objective functions required to maximize the generalized benefit of both parties are described. Section 3 presents the solutions of differential game relationships in three scenarios, including the cooperative, non-cooperative and Stackelberg mode. Section 4 compares the results, and the numerical simulation is conducted in Section 5. The game results with different model parameters are further explored in Section 6. Finally, the conclusions are presented in Section 7.

## 2. Problem Formulation

The assumption is made that both the supervisor and postgraduate are rational decision-makers. The supervisor’s and postgraduate’s behavior in their academic activities can be divided into positive and negative efforts, which are effectively the evolutionary dynamics of their relationship. Additionally, the supervisor–postgraduate academic community can be viewed as a dynamic system. Then, the efforts of both the supervisor and postgraduate can be regarded as the control variables of the system, and the academic level of the community can be regarded as the state variable of the system. The supervisor’s positive efforts include, for example, the active academic guidance, spiritual encouragement and mentoring provided to the postgraduate, as well as the improvement of the scientific research conditions. The negative efforts of the supervisor include, for example, arranging non-academic affairs for the postgraduate and even compelling them to act as cheap labor for private profit. Other examples could be spiritual discouragement and threatening the postgraduate. The postgraduate’s positive efforts are the active and highly efficient devotion to the academic research, while the negative efforts could include various passive and inefficient researching behaviors, such as showing up for work but not exerting much effort.

The differential equation of the general system can be described as follows:

(1) $$\dot{x}(t)=f(x,t)$$

where x is the state variable, $\dot{x}$ is the derivative of x, t is the time variable and f(·) is the evolutionary dynamics of the state variable.

The academic level of the supervisor–postgraduate community is related to their positive and negative efforts. Then, the differential equation of the state variable can be expressed as follows:

(2) $$\dot{x}(t)=\alpha_{t,p}E_{t,p}(t)+\alpha_{s,p}E_{s,p}(t)-\alpha_{t,n}E_{t,n}(t)-\alpha_{s,n}E_{s,n}(t)-\delta x(t)$$

where the state variable x is the academic level of the supervisor–postgraduate community, $\dot{x}$ is the improvement or setback of the academic level, $E_{t,p}$ and $E_{t,n}$ are the supervisor’s positive and negative efforts, respectively; $\alpha_{t,p}$ and $\alpha_{t,n}$ are the influencing coefficients of the supervisor’s positive and negative efforts on the academic level, respectively; $E_{s,p}$ and $E_{s,n}$ are the postgraduate’s positive and negative efforts, respectively; $\alpha_{s,p}$ and $\alpha_{s,n}$ are the influencing coefficients of the postgraduate’s positive and negative efforts on the academic level, respectively, and δ is the decay factor, which reflects the natural decay of the academic level over time. The terms $\alpha_{t,p}E_{t,p}(t)$ and $\alpha_{s,p}E_{s,p}(t)$ denote the improvement of the academic level of the community caused by the positive efforts of the supervisor and postgraduate, respectively; the terms $\alpha_{t,n}E_{t,n}(t)$ and $\alpha_{s,n}E_{s,n}(t)$ denote the decline of the academic level of the community caused by the supervisor and postgraduate’s negative efforts, respectively.

The efforts of the supervisor and postgraduate can bring about academic benefits, but those benefits also come with costs. Here, the costs of both actors are described by using the quadratic function:

(3a) $${Cost}_{t}=\frac{1}{2}c_{t,p}E_{t,p}^{2}+\frac{1}{2}c_{t,n}E_{t,n}^{2}$$

(3b) $${Cost}_{s}=\frac{1}{2}c_{s,p}E_{s,p}^{2}+\frac{1}{2}c_{s,n}E_{s,n}^{2}$$

where ${Cost}_{t}$ and ${Cost}_{s}$ are the costs of the supervisor and postgraduate, respectively; $c_{t,p}$ and $c_{t,n}$ are the cost coefficients of the supervisor’s positive and negative efforts, respectively, and $c_{s,p}$ and $c_{s,n}$ are the cost coefficients of the postgraduate’s positive and negative efforts, respectively.

When the supervisor and postgraduate exert different levels of types of efforts, they can obtain different benefits. Here, the supervisor’s benefit is the generalized one, including the benefits from the positive efforts, such as the improvement of the academic level of the community, and the improved social reputation due to the supervisor’s excellent coaching ability. Additionally, the benefits from the negative efforts are included, such as the profits from non-academic affairs. For example, some supervisors may require a postgraduate to conduct scientific projects that are unrelated to the postgraduate’s research topic, but which can bring benefits to the supervisor, and some supervisors even treat postgraduates as cheap and lowly employees in their enterprises. As a result, the benefit function of the supervisor can be expressed as:

(4) $$J_{t}=\int_{0}^{T}e^{-\rho t}\left(\lambda_{t}x(t)+k_{t,p}E_{t,p}(t)+k_{t,n}E_{t,n}(t)-{Cost}_{s}(t)\right)dt$$

where $J_{t}$ is the total benefit of the supervisor, T is the duration of the supervisor-postgraduate relationship, t is the time variable, ρ is the discount rate, $\lambda_{t}$ is the efficiency coefficient with regard to the change of the community’s academic level and $k_{t,p}$ and $k_{t,n}$ are the benefit coefficients of the supervisor’s positive and negative efforts, respectively. The terms $\lambda_{t}x(t)$, $k_{t,p}E_{t,p}(t)$ and $k_{t,n}E_{t,n}(t)$ are the instantaneous benefit from the academic level change, and the supervisor’s positive and negative efforts, respectively. The term ${Cost}_{s}(t)$ is the supervisor’s instantaneous cost caused by the positive and negative efforts.

The generalized benefit of the postgraduate includes the direct benefits accruing from the improvement of the academic level, as well as the enhancement of self-worth and personal character in conducting research. Then, the benefit function of the postgraduate can be described as:

(5) $$J_{s}=max\int_{0}^{T}e^{-\rho t}\left(\lambda_{s}x(t)+k_{s,p}E_{s,p}(t)+k_{s,n}E_{s,n}(t)-{Cost}_{s}(t)\right)dt$$

where $J_{s}$ is the total benefit of the postgraduate, $\lambda_{s}$ is the postgraduate’s efficiency coefficient with regard to the improvement of academic level and $k_{s,p}$ and $k_{s,n}$ are the benefit coefficients of the postgraduate’s positive and negative efforts, respectively. The terms $\lambda_{s}x(t)$, $k_{s,p}E_{s,p}(t)$ and $k_{s,n}E_{s,n}(t)$ are the instantaneous benefit from the change of academic level of the community, and the supervisor’s positive and negative efforts, respectively. The term ${Cost}_{s}(t)$ is the supervisor’s instantaneous cost caused by the positive and negative efforts.

## 3. Game Model and Solutions in Three Scenarios

In this sector, the game relationship between the supervisor and postgraduates is formulated and solved in the non-cooperative, cooperative and Stackelberg scenarios.

### 3.1. Non-Cooperative Scenario

In the non-cooperative scenario, both the supervisor and the postgraduate make efforts to maximize their respective generalized benefits, but without considering the interests of the other party. Then, the objective functions can be described as follows:

(6a) $$J_{t}=max\int_{0}^{t}e^{-\rho t}\left(\lambda_{t}x+k_{t,p}E_{t,p}+k_{t,n}E_{t,n}-\frac{1}{2}c_{t,p}E_{t,p}^{2}-\frac{1}{2}c_{t,n}E_{t,n}^{2}\right)dt$$

(6b) $$J_{s}=max\int_{0}^{t}e^{-\rho t}\left(\lambda_{s}x+k_{s,p}E_{s,p}+k_{s,n}E_{s,n}-\frac{1}{2}c_{s,p}E_{s,p}^{2}-\frac{1}{2}c_{s,n}E_{s,n}^{2}\right)dt$$

For this game mode, the optimal equilibrium strategy can be solved by employing Hamilton–Jacobi–Bellman (HJB) equations [44]. The HJB equations of both the supervisor and postgraduate can be formulated as:

(7a) $$\rho V_{t}^{N}=max\{\lambda_{t}x+k_{t,p}E_{t,p}+k_{t,n}E_{t,n}-\frac{1}{2}c_{t,p}E_{t,p}^{2}-\frac{1}{2}c_{t,n}E_{t,n}^{2}+{\dot{V}}_{t}^{N}\left(\alpha_{t,p}E_{t,p}+\alpha_{s,p}E_{s,p}-\alpha_{t,n}E_{t,n}-\alpha_{s,n}E_{s,n}-\delta x\right)\}$$

(7b) $$\rho V_{s}^{N}=max\{\lambda_{s}x+k_{s,p}E_{s,p}+k_{s,n}E_{s,n}-\frac{1}{2}c_{s,p}E_{s,p}^{2}-\frac{1}{2}c_{s,n}E_{s,n}^{2}+{\dot{V}}_{s}^{N}\left(\alpha_{t,p}E_{t,p}+\alpha_{s,p}E_{s,p}-\alpha_{t,n}E_{t,n}-\alpha_{s,n}E_{s,n}-\delta x\right)\}$$

where $V_{t}^{N}$ and $V_{s}^{N}$ are the optimal benefit function of the supervisor and postgraduate, respectively. The superscript *N* denotes the non-cooperative game. In the following sections, the superscripts *C* and *S* will denote the cooperative game and Stackelberg game modes, respectively. The variable with the specific superscript corresponds to the game mode. For example, $E_{t,p}^{N}$ denotes the supervisor’s positive efforts in the non-cooperative mode, $E_{s,n}^{C}$ denotes the postgraduate’s negative efforts in the cooperative game and $x^{S}$ denotes the academic level of the community in the Stackelberg game.

Differentiating Equation (7a,b) with respect to $E_{t,p}$, $E_{t,n}$ and $E_{s,p}$, $E_{s,n}$, respectively, and setting to 0, we obtain:

(8a) $$E_{t,p}^{N}=\frac{k_{t,p}+{\dot{V}}_{t}^{N}\alpha_{t,p}}{c_{t,p}}$$

(8b) $$E_{t,n}^{N}=\frac{k_{t,n}-{\dot{V}}_{t}^{N}\alpha_{t,n}}{c_{s,n}}$$

(8c) $$E_{s,p}^{N}=\frac{k_{s,p}+{\dot{V}}_{s}^{N}\alpha_{s,p}}{c_{s,p}}$$

(8d) $$E_{s,n}^{N}=\frac{k_{s,n}-{\dot{V}}_{s}^{N}\alpha_{s,n}}{c_{s,n}}$$

Substituting Equation (8a–d) into the HJB equations, we obtain:

(9a) $$\rho V_{t}^{N}=\lambda_{t}x+k_{t,p}(\frac{k_{t,p}+{\dot{V}}_{t}^{N}\alpha_{t,p}}{c_{t,p}})+k_{t,n}(\frac{k_{t,n}-{\dot{V}}_{t}^{N}\alpha_{t,n}}{c_{t,n}})-\frac{1}{2}c_{t,p}{\left(\frac{k_{t,p}+{\dot{V}}_{t}^{N}\alpha_{t,p}}{c_{t,p}}\right)}^{2}-\frac{1}{2}c_{t,n}{\left(\frac{k_{t,n}-{\dot{V}}_{t}^{N}\alpha_{t,n}}{c_{t,n}}\right)}^{2}\;+{\dot{V}}_{t}^{N}\left(\alpha_{t,p}(\frac{k_{t,p}+{\dot{V}}_{t}^{N}\alpha_{t,p}}{c_{t,p}})+\alpha_{s,p}(\frac{k_{s,p}+{\dot{V}}_{s}^{N}\alpha_{s,p}}{c_{s,p}})-\alpha_{t,n}(\frac{k_{t,n}-{\dot{V}}_{t}^{N}\alpha_{t,n}}{c_{t,n}})-\alpha_{s,n}(\frac{k_{s,n}-{\dot{V}}_{s}^{N}\alpha_{s,n}}{c_{s,n}})-\delta x\right)$$

(9b) $$\rho V_{s}^{N}=\lambda_{s}x+k_{s,p}(\frac{k_{s,p}+{\dot{V}}_{s}^{N}\alpha_{s,p}}{c_{s,p}})+k_{s,n}(\frac{k_{s,n}-{\dot{V}}_{s}^{N}\alpha_{s,n}}{c_{s,n}})-\frac{1}{2}c_{s,p}{\left(\frac{k_{s,p}+{\dot{V}}_{s}^{N}\alpha_{s,p}}{c_{s,p}}\right)}^{2}-\frac{1}{2}c_{s,n}{\left(\frac{k_{s,n}-{\dot{V}}_{s}^{N}\alpha_{s,n}}{c_{s,n}}\right)}^{2}\;+{\dot{V}}_{s}^{N}\left(\alpha_{t,p}(\frac{k_{t,p}+{\dot{V}}_{t}^{N}\alpha_{t,p}}{c_{t,p}})+\alpha_{s,p}(\frac{k_{s,p}+{\dot{V}}_{s}^{N}\alpha_{s,p}}{c_{s,p}})-\alpha_{t,n}(\frac{k_{t,n}-{\dot{V}}_{t}^{N}\alpha_{t,n}}{c_{t,n}})-\alpha_{s,n}(\frac{k_{s,n}-{\dot{V}}_{s}^{N}\alpha_{s,n}}{c_{s,n}})-\delta x\right)$$

According to the structure of Equation (9a,b), the linear optimal benefit function of $x\left(t\right)$ is the solution to the HJB equations:

(10a) $$V_{t}^{N}=A_{1}^{N}x\left(t\right)+B_{1}^{N}$$

(10b) $$V_{s}^{N}=A_{2}^{N}x\left(t\right)+B_{2}^{N}$$

where $A_{1}^{N}$, $B_{1}^{N}$, $A_{2}^{N}$ and $B_{2}^{N}$ are constants.

Differentiating Equation (10a,b) with respect to x, we obtain:

$${\dot{V}}_{t}^{N}=A_{1}^{N}$$

$${\dot{V}}_{s}^{N}=A_{2}^{N}$$

Substituting $V_{t}^{N}$, ${\dot{V}}_{t}^{N}$, ${\dot{V}}_{t}^{N}$ and ${\dot{V}}_{s}^{N}$ into Equation (9a,b), we obtain:

$$A_{1}^{N}=\frac{\lambda_{t}}{\rho +\delta }$$

$$A_{2}^{N}=\frac{\lambda_{s}}{\rho +\delta }$$

$$B_{1}^{N}=\frac{{\left(k_{t,p}+\frac{\lambda_{t}\alpha_{t,p}}{\rho +\delta }\right)}^{2}}{2\rho c_{t,p}}+\frac{{\left(k_{t,n}-\frac{\lambda_{t}\alpha_{t,n}}{\rho +\delta }\right)}^{2}}{2\rho c_{t,n}}+\frac{\lambda_{t}k_{s,p}\alpha_{s,p}+\frac{\lambda_{t}\lambda_{s}\alpha_{s,p}^{2}}{\rho +\delta }}{\rho c_{s,p}(\rho +\delta )}-\frac{\lambda_{t}k_{s,n}\alpha_{s,n}-\frac{\lambda_{t}\lambda_{s}\alpha_{s,n}^{2}}{\rho +\delta }}{\rho c_{s,n}(\rho +\delta )}$$

$$B_{2}^{N}=\frac{{\left(k_{s,p}+\frac{\lambda_{s}\alpha_{s,p}}{\rho +\delta }\right)}^{2}}{2\rho c_{s,p}}+\frac{{\left(k_{s,n}-\frac{\lambda_{s}\alpha_{s,n}}{\rho +\delta }\right)}^{2}}{2\rho c_{s,n}}+\frac{\lambda_{s}k_{t,p}\alpha_{t,p}+\frac{\lambda_{t}\lambda_{s}\alpha_{t,p}^{2}}{\rho +\delta }}{\rho c_{t,p}(\rho +\delta )}-\frac{\lambda_{s}k_{t,n}\alpha_{t,n}-\frac{\lambda_{t}\lambda_{s}\alpha_{t,n}^{2}}{\rho +\delta }}{\rho c_{t,n}\left(\rho +\delta \right)}$$

Therefore, the optimal equilibrium strategies for both the supervisor and postgraduate are as follows:

$$E_{t,p}^{N}=\frac{k_{t,p}+\frac{\lambda_{t}}{\rho +\delta }\alpha_{t,p}}{c_{t,p}},\;E_{t,n}^{N}=\frac{k_{t,n}-\frac{\lambda_{t}}{\rho +\delta }\alpha_{t,n}}{c_{t,n}},\;E_{s,p}^{N}=\frac{k_{s,p}+\frac{\lambda_{s}}{\rho +\delta }\alpha_{s,p}}{c_{s,p}},\;E_{s,n}^{N}=\frac{k_{s,n}-\frac{\lambda_{s}}{\rho +\delta }\alpha_{s,n}}{c_{s,n}}$$

The trajectory of the optimal benefit of both the supervisor and the postgraduate can be obtained as:

$$V_{t}^{N}=A_{1}^{N}x+B_{1}^{N}$$

$$V_{s}^{N}=A_{2}^{N}x+B_{2}^{N}$$

The trajectory of the optimal academic level of the community can be obtained as:

$$x^{N}\left(t\right)=e^{-\delta t}\left(x_{0}-\frac{Y^{N}}{\delta }\right)+\frac{Y^{N}}{\delta }$$

where $Y^{N}=\alpha_{t,p}\frac{k_{t,p}+\frac{\lambda_{t}}{\rho +\delta }\alpha_{t,p}}{c_{t,p}}+\alpha_{t,p}\frac{k_{t,p}+\frac{\lambda_{s}}{\rho +\delta }\alpha_{t,p}}{c_{t,p}}-\alpha_{t,n}\frac{k_{t,n}-\frac{\lambda_{t}}{\rho +\delta }\alpha_{t,n}}{c_{t,n}}-\alpha_{s,n}\frac{k_{s,n}-\frac{\lambda_{s}}{\rho +\delta }\alpha_{s,n}}{c_{s,n}}$.

### 3.2. Cooperative Scenario

In this game scenario, both the supervisor and postgraduate collaboratively make efforts to maximize the benefit of the academic community. Then, the objective function for the cooperative mode can be expressed as:

(11) $$J^{C}=max\int_{0}^{T}e^{-\rho t}\left(\lambda_{t}x+k_{t,p}E_{t,p}+k_{t,n}E_{t,n}+\lambda_{s}x+k_{s,p}E_{s,p}+k_{s,n}E_{s,n}-\frac{1}{2}c_{t,p}E_{t,p}^{2}-\frac{1}{2}c_{t,n}E_{t,n}^{2}-\frac{1}{2}c_{s,p}E_{s,p}^{2}-\frac{1}{2}c_{s,n}E_{s,n}^{2}\right)dt$$

where $J^{C}$ is the total benefit of the community over the duration of their relationship.

For the cooperative game mode, the optimal solution can be obtained by constructing the following HJB equation:

(12) $$\rho V^{C}=max\{\left[\lambda_{t}x+k_{t,p}E_{t,p}+k_{t,n}E_{t,n}+\lambda_{s}x+k_{s,p}E_{s,p}+k_{s,n}E_{s,n}-\frac{1}{2}c_{t,p}E_{t,p}^{2}-\frac{1}{2}c_{t,n}E_{t,n}^{2}-\frac{1}{2}c_{s,p}E_{s,p}^{2}-\frac{1}{2}c_{s,n}E_{s,n}^{2}\right]+\;{\dot{V}}^{C}\left(\alpha_{t,p}E_{t,p}+\alpha_{s,p}E_{s,p}-\alpha_{t,n}E_{t,n}-\alpha_{s,n}E_{s,n}-\delta x\right)\}$$

Differentiating Equation (12) with respect to $E_{t,p}$, $E_{t,n}$ and $E_{s,p}$, $E_{s,n}$, respectively, and setting to 0, the optimal equilibrium strategy of the efforts can be solved as follows: (for the detailed derivation, refer to Section 3.1)

$$E_{t,p}^{C}=\frac{k_{t,p}+\frac{\lambda_{t}+\lambda_{s}}{\rho +\delta }\alpha_{t,p}}{c_{t,p}},\;E_{t,n}^{C}=\frac{k_{t,n}-\frac{\lambda_{t}+\lambda_{s}}{\rho +\delta }\alpha_{t,n}}{c_{t,n}},\;E_{s,p}^{C}=\frac{k_{s,p}+\frac{\lambda_{t}+\lambda_{s}}{\rho +\delta }\alpha_{s,p}}{c_{s,p}},\;E_{s,n}^{C}=\frac{k_{s,n}-\frac{\lambda_{t}+\lambda_{s}}{\rho +\delta }\alpha_{s,n}}{c_{s,n}}$$

Then, the optimal benefit function of both the supervisor and postgraduate can be calculated as:

$$V^{C}=A^{C}x+B^{C}$$

where $A^{C}=\frac{\lambda_{t}+\lambda_{s}}{\rho +\delta }$, and $B^{C}=\frac{{\left(k_{t,p}+\frac{\lambda_{t}+\lambda_{s}}{\rho +\delta }\alpha_{t,p}\right)}^{2}}{{2\rho c}_{t,p}}+\frac{{\left(k_{t,n}-\frac{\lambda_{t}+\lambda_{s}}{\rho +\delta }\alpha_{t,n}\right)}^{2}}{{2\rho c}_{t,n}}+\frac{{\left(k_{s,p}+\frac{\lambda_{t}+\lambda_{s}}{\rho +\delta }\alpha_{s,p}\right)}^{2}}{{2\rho c}_{s,p}}+\frac{{\left(k_{s,n}-\frac{\lambda_{t}+\lambda_{s}}{\rho +\delta }\alpha_{s,n}\right)}^{2}}{{2\rho c}_{s,n}}$.

The optimal trajectory of the optimal academic level of the community can be calculated as:

$$x^{C}\left(t\right)=e^{-\delta t}\left(x_{0}-\frac{Y^{C}}{\delta }\right)+\frac{Y^{C}}{\delta }$$

where $x_{0}$ is the initial value of the state variable x, and $Y^{C}=\alpha_{t,p}\frac{k_{t,p}+\frac{\lambda_{t}+\lambda_{s}}{\rho +\delta }\alpha_{t,p}}{c_{t,p}}+\alpha_{s,p}\frac{k_{s,p}+\frac{\lambda_{t}+\lambda_{s}}{\rho +\delta }\alpha_{s,p}}{c_{s,p}}-\alpha_{t,n}\frac{k_{t,n}-\frac{\lambda_{t}+\lambda_{s}}{\rho +\delta }\alpha_{t,n}}{c_{t,n}}-\alpha_{s,n}\frac{k_{s,n}-\frac{\lambda_{t}+\lambda_{s}}{\rho +\delta }\alpha_{s,n}}{c_{s,n}}$.

### 3.3. Stackelberg Scenario

Unlike the cooperative and non-cooperative game scenarios, the Stackelberg game is a supervisor-led process. In this game mode, the postgraduate passively makes decisions according to the supervisor’s behaviors. The game can be divided into two stages. In the first stage, the supervisor makes efforts and shares part of the cost for the benefit of the postgraduate. Examples of sharing the cost could include the supervisor providing a certain amount of economic assistance and/or the spiritual encouragement to the postgraduate, and/or the supervisor could improve the scientific research conditions for the postgraduate. In the second stage, the postgraduate makes a decision with regard to his or her own efforts based on the supervisor’s decision. Then, the objective function of both actors can be formulated as:

$$J_{t}=max\int_{0}^{T}e^{-\rho t}\left(\lambda_{t}x+k_{t,p}E_{t,p}+k_{t,n}E_{t,n}-\frac{1}{2}c_{t,p}E_{t,p}^{2}-\frac{1}{2}c_{t,n}E_{t,n}^{2}-i(\frac{1}{2}c_{s,p}E_{s,p}^{2}+\frac{1}{2}c_{s,n}E_{s,n}^{2})\right)dt$$

$$J_{s}=max\int_{0}^{T}e^{-\rho t}\left(\lambda_{s}x+k_{s,p}E_{s,p}+k_{s,n}E_{s,n}-(1-i)(\frac{1}{2}c_{s,p}E_{s,p}^{2}+\frac{1}{2}c_{s,n}E_{s,n}^{2})\right)dt$$

where i is the supervisor’s share ratio of the cost for the postgraduate.

Similar to the solution in the non-cooperative and cooperative game scenarios, the optimal equilibrium strategy of efforts for both the supervisor and postgraduate can be solved by constructing an HJB equation (for the detailed derivation, refer to Section 3.1):

$$E_{t,p}^{S}=\frac{k_{t,p}+\frac{\lambda_{t}}{\rho +\delta }\alpha_{t,p}}{c_{t,p}}$$

$$E_{t,n}^{S}=\frac{k_{t,n}-\frac{\lambda_{t}}{\rho +\delta }\alpha_{t,n}}{c_{t,n}}$$

$$E_{s,p}^{S}=\frac{k_{s,p}+\frac{\lambda_{s}}{\rho +\delta }\alpha_{s,p}}{(1-i)c_{s,p}}$$

$$E_{s,n}^{S}=\frac{k_{s,n}-\frac{\lambda_{s}}{\rho +\delta }\alpha_{s,n}}{(1-i)c_{s,n}}$$

The optimal benefit function of both actors can be obtained as:

$$V_{t}^{S}=A_{1}^{S}x+B_{1}^{S}$$

$$V_{s}^{S}=A_{2}^{S}x+B_{2}^{S}$$

where $A_{1}^{S}=\frac{\lambda_{t}}{\rho +\delta }$, $A_{2}^{S}=\frac{\lambda_{s}}{\rho +\delta }$, $B_{1}^{S}=\frac{{\left(k_{t,p}+\frac{\lambda_{t}}{\rho +\delta }\alpha_{t,p}\right)}^{2}}{{2\rho c}_{t,p}}+\frac{{\left(k_{t,n}-\frac{\lambda_{t}}{\rho +\delta }\alpha_{t,n}\right)}^{2}}{{2\rho c}_{t,n}}-\frac{i}{2\rho }(c_{s,p}{\left(\frac{k_{s,p}+\frac{\lambda_{s}}{\rho +\delta }\alpha_{s,p}}{\left(1-i\right)c_{s,p}}\right)}^{2}-c_{s,n}{\left(\frac{k_{s,n}-\frac{\lambda_{s}}{\rho +\delta }\alpha_{s,n}}{{\left(1-i\right)c}_{s,n}}\right)}^{2})+\frac{\lambda_{t}k_{s,p}\alpha_{s,p}+\frac{\lambda_{t}\lambda_{s}\alpha_{s,p}^{2}}{\rho +\delta }}{\left(1-i\right)\rho c_{s,p}(\rho +\delta )}-\frac{\lambda_{t}k_{s,n}\alpha_{s,n}-\frac{\lambda_{t}\lambda_{s}\alpha_{s,n}^{2}}{\rho +\delta }}{\left(1-i\right)\rho c_{s,n}(\rho +\delta )}$, and $B_{2}^{S}=\frac{{\left(k_{s,p}+\frac{\lambda_{s}}{\rho +\delta }\alpha_{s,p}\right)}^{2}}{2\rho (1-i)c_{s,p}}+\frac{{\left(k_{s,n}-\frac{\lambda_{s}}{\rho +\delta }\alpha_{s,n}\right)}^{2}}{2\rho (1-i)c_{s,n}}+\frac{\lambda_{s}k_{t,p}\alpha_{t,p}+\frac{\lambda_{t}\lambda_{s}\alpha_{t,p}^{2}}{\rho +\delta }}{\rho c_{t,p}(\rho +\delta )}-\frac{\lambda_{s}k_{t,n}\alpha_{t,n}-\frac{\lambda_{t}\lambda_{s}\alpha_{t,n}^{2}}{\rho +\delta }}{\rho c_{t,n}(\rho +\delta )}$.

The trajectory of the optimal academic level of the community can be calculated as:

$$x^{S}\left(t\right)=e^{-\delta t}\left(x_{0}-\frac{Y^{S}}{\delta }\right)+\frac{Y^{S}}{\delta }$$

where $Y^{S}=\alpha_{t,p}\frac{k_{t,p}+\frac{\lambda_{t}}{\rho +\delta }\alpha_{t,p}}{c_{t,p}}+\alpha_{s,p}\frac{k_{s,p}+\frac{\lambda_{s}}{\rho +\delta }\alpha_{s,p}}{(1-i)c_{s,p}}-\alpha_{t,n}\frac{k_{t,n}-\frac{\lambda_{t}}{\rho +\delta }\alpha_{t,n}}{c_{t,n}}-\alpha_{s,n}\frac{k_{s,n}-\frac{\lambda_{s}}{\rho +\delta }\alpha_{s,n}}{(1-i)c_{s,n}}$.

## 4. Comparison of Equilibrium Strategies

In this section, several conclusions can be obtained by comparing the equilibrium strategies generated in the non-cooperative, cooperative and Stackelberg game scenarios.

### 4.1. Comparison of Efforts

By comparing the optimal equilibrium solutions in different game scenarios, the relationship of the positive and negative efforts for both the supervisor and postgraduate are obtained as follows:

$$E_{t,p}^{C}>E_{t,p}^{S}=E_{t,p}^{N}$$

$$E_{t,n}^{C}<E_{t,n}^{S}=E_{t,n}^{N}$$

$$E_{s,n}^{S}>E_{s,n}^{N}>E_{s,n}^{C}$$

$$E_{s,p}^{N}<E_{s,p}^{C}$$

$$E_{s,p}^{N}<E_{s,p}^{S}$$

$$E_{s,p}^{C}-E_{s,p}^{S}=\frac{\lambda_{t}\alpha_{s,p}-i(\left(\rho +\delta \right)k_{s,p}+(\lambda_{t}+\lambda_{s})\alpha_{s,p})}{\left(1-i)c_{s,p}(\rho +\delta \right)}$$

One can easily know that when $i<\frac{\lambda_{t}\alpha_{s,p}}{\left(\rho +\delta \right)k_{s,p}+(\lambda_{t}+\lambda_{s})\alpha_{s,p}}$, $E_{s,p}^{C}>E_{s,p}^{S}$. The comparison indicates that the supervisor’s positive and negative efforts for both the non-cooperative and Stackelberg game are equal. The supervisor’s positive effort for the cooperative game is higher than the efforts for the non-cooperative and Stackelberg games, while the supervisor’s negative effort for the cooperative game is less than the other two game modes. This is consistent with the fact that when both game actors conduct the scientific research in the cooperative mode, the supervisor is more willing to devote their efforts to mentoring the postgraduates and improving the academic level.

The negative effort of the postgraduate is the highest in the Stackelberg game and the lowest in the cooperative game. For the Stackelberg game, if $i<\frac{\lambda_{t}\alpha_{s,p}}{\left(\rho +\delta \right)k_{s,p}+(\lambda_{t}+\lambda_{s})\alpha_{s,p}}$, the positive effort of the postgraduate increases in line with the growing share ratio of the cost, and its Pareto optimality tends to the optimal equilibrium strategy of the positive efforts in the cooperative game. This implies that the postgraduate is also more willing to devote his or her efforts to the academic activity in the cooperative game. For the Stackelberg game, however, the positive effort of the postgraduate increases in line with the growing sharing ratio, even exceeding that of the non-cooperative game. Moreover, because the growth rate of the postgraduate’s benefit from the improvement of the positive effort declines, whereas the growth rate of the postgraduate’s benefit from the improvement of the negative effort still increases, the negative effort of the postgraduate may increase as the postgraduate aims to improve the individual benefit. Therefore, it is critical to control the supervisor’s sharing ratio to within a reasonable level, in order to realize an appropriate balance between the postgraduate’s positive and negative efforts.

### 4.2. Comparison of Academic Level of the Community

Here, x (the academic level of supervisor–postgraduate community) and Y (a temporary variable defined to express the function of x) are positively correlated and are firstly explained, and then, x and Y are compared in the three game modes.

Since $x\left(t\right)=e^{-\delta t}\left(x_{0}-\frac{Y}{\delta }\right)+\frac{Y}{\delta }$, then $\frac{dx}{dY}=\frac{\left(1-e^{-\delta t}\right)}{\delta }$. As δ and t are both positive numbers; thus, $e^{-\delta t}<1$. It is easy to know that $\frac{dx}{dY}>0$. Therefore, x and Y are positively correlated. That is to say, the greater the Y is, the greater the x.

As ${Y^{C}-Y}^{S}=\frac{\frac{\lambda_{s}}{\rho +\delta }\alpha_{t,p}^{2}}{c_{t,p}}+\frac{\frac{\lambda_{s}}{\rho +\delta }\alpha_{t,n}^{2}}{c_{t,n}}+\frac{\frac{\lambda_{t}}{\rho +\delta }\alpha_{s,p}^{2}}{c_{s,p}}-\frac{i}{\left(1-i\right)}\alpha_{s,p}\frac{k_{s,p}+\frac{\lambda_{s}}{\rho +\delta }\alpha_{s,p}}{c_{s,p}}-\frac{\frac{\lambda_{t}}{\rho +\delta }\alpha_{s,n}^{2}}{c_{s,n}}+\frac{i}{\left(1-i\right)}\alpha_{s,n}\frac{k_{s,n}+\frac{\lambda_{s}}{\rho +\delta }\alpha_{s,n}}{c_{s,n}}>0$, and ${Y^{C}-Y}^{N}=\frac{\frac{\lambda_{s}}{\rho +\delta }\alpha_{t,p}^{2}}{c_{t,p}}+\frac{\frac{\lambda_{t}}{\rho +\delta }\alpha_{s,p}^{2}}{c_{s,p}}+\frac{\frac{\lambda_{s}}{\rho +\delta }\alpha_{t,n}^{2}}{c_{t,n}}+\frac{\frac{\lambda_{t}}{\rho +\delta }\alpha_{s,n}^{2}}{c_{s,n}}>0$,

Then,

(13) $${Y^{S}-Y}^{N}=\frac{i}{\left(1-i\right)}(\alpha_{s,p}\frac{k_{s,p}+\frac{\lambda_{s}}{\rho +\delta }\alpha_{s,p}}{c_{s,p}}-\alpha_{s,n}\frac{k_{s,n}-\frac{\lambda_{s}}{\rho +\delta }\alpha_{s,n}}{c_{s,n}})$$

For Equation (13), if the coefficients $c_{s,p}$ and $c_{s,n}$ and $\alpha_{s,p}k_{s,p}$ and $\alpha_{s,n}k_{s,n}$ are similar, respectively, the ${Y^{S}-Y}^{N}>0$. Then, one can obtain that:

$${Y^{C}>Y}^{S}>Y^{N}$$

According to the positive correlation between x and Y, one can also obtain that:

$$x^{C}>x^{S}>x^{N}$$

The results demonstrate that the optimal academic level of the supervisor–postgraduate community for the cooperative game was the highest, followed by the Stackelberg game, and finally by the non-cooperative game. This is because, for the cooperative game, the supervisor and postgraduate have the common goal of maximizing the total benefit of the community, which can inspire both of them to make great efforts to improve their academic level. Therefore, the academic level in this game was higher than that in the non-cooperative game.

### 4.3. Comparison of the Community’s Optimal Total Benefit

The relationship of the community’s optimal total benefit is as follows:

$$V^{C}>V^{S}>V^{N}$$

The community’s optimal total benefit (sum of the optimal benefit of the supervisor and that of the postgraduate) for the cooperative game is the highest, followed by the Stackelberg mode, and then the non-cooperative mode. Inspired by having a common purpose in the cooperative game, both the supervisor and the postgraduate strive for the total benefit of the community, and thus, they obtain the maximum benefit. For the Stackelberg game, as the supervisor shares a part of the cost with the postgraduate, the postgraduate’s motivation can be somewhat spurred on, and they will make more positive efforts in the research work. Therefore, the optimal total benefit in the Stackelberg game mode is also higher than that in the non-cooperative game.

## 5. Numerical Simulation and Analysis

In this section, a numerical simulation was conducted to quantitatively discuss the critical indicators in the three game scenarios.

### 5.1. Parameter Settings

The parameters were set as follows: $c_{t,p}$ = 1.5, $c_{t,n}$ = 1.5, $c_{s,p}$ = 1.5, $c_{s,n}$ = 1.5, $\alpha_{t,p}$ = 0.6, $\alpha_{t,n}$ = 0.4, $\alpha_{s,p}$ = 0.5, $\alpha_{s,n}$ = 0.4, $\lambda_{t}$ = 0.5, $\lambda_{s}$ = 0.4, $k_{t,p}$ = 0.5, $k_{t,n}$ = 0.4, $k_{s,p}$ = 0.5, $k_{s,n}$ = 0.4, ρ = 0.9, δ = 0.2, i = 0.4, $x_{0}$ = 1, $\eta_{t}$ = 0.5 and $\eta_{s}$ = 0.5.

### 5.2. Results and Analysis

#### 5.2.1. Quantitative Analysis

Table 1 summarizes the results of the optimal equilibrium strategies in the three game modes. Compared to the cooperative game, the non-cooperative game can bring about the worst effect for both actors. Specifically, compared to the results in the cooperative game, in the non-cooperative game, the supervisor’s positive efforts were reduced by 22.01%, while the negative effort was increased by up to 200%. The postgraduate’s positive effort was reduced by 25.01%, while the negative effort was raised by up to 249.9%. Thereby, the optimal academic level and total benefit of the community were decreased by 37.90% and 32.01%, respectively.

Compared to the non-cooperative game, in the Stackelberg scenario, the supervisor’s positive and negative efforts remained unchanged, while the postgraduate’s positive and negative efforts increased by 66.69% and 66.64%, respectively. In addition, the academic level was raised by 25.90%, thereby contributing to a 19.80% improvement in the optimal total benefit. That is to say, the optimal total benefits of both actors were improved in the Stackelberg mode.

Compared to the Stackelberg game, in the cooperative game, the supervisor’s positive effort was increased by 28.22%, while the negative effort was reduced by 66.67%. Conversely, the postgraduate’s positive and negative efforts were reduced by 20% and 82.85%, respectively. Thereby, the optimal academic level was raised by 27.88%, and the optimal total benefit of the community was increased by 22.78%. Similar to the results of the Stackelberg game, both the supervisor’s and postgraduate’s benefits were improved in the cooperative game.

#### 5.2.2. Graphic Analysis

**Figure 1:**
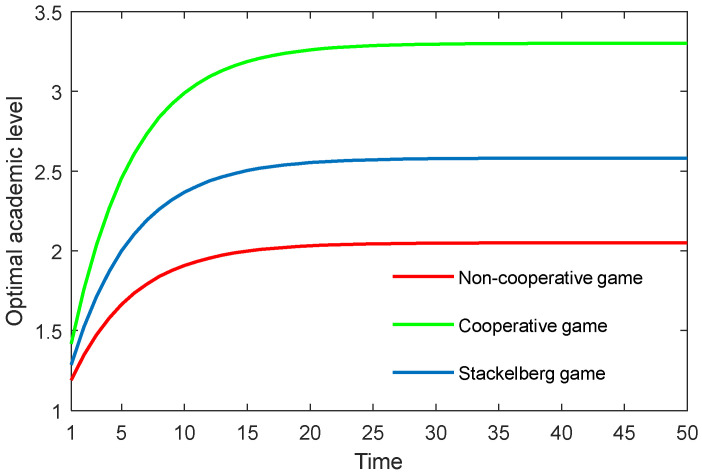
Optimal academic level of the community over time in three game scenarios.

**Figure 2:**
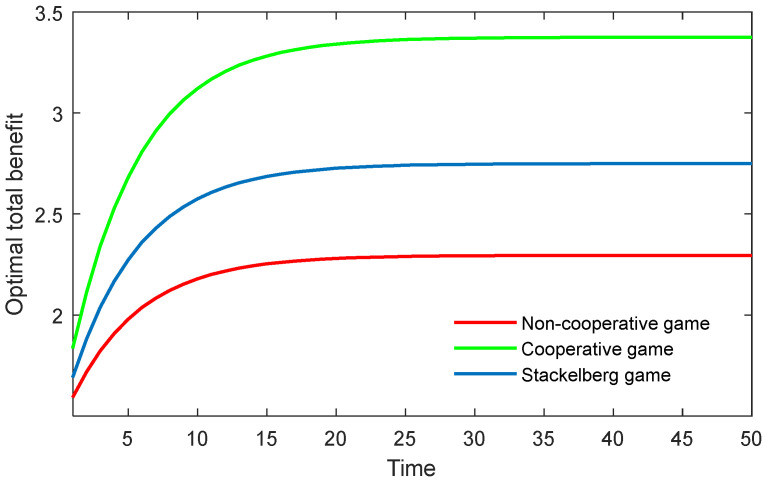
Optimal total benefit of both the supervisor and postgraduate over time in three game scenarios.

**Figure 3:**
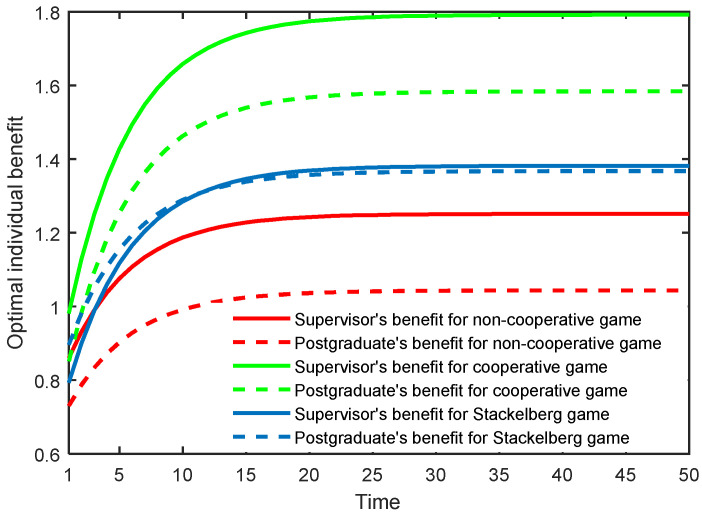
Optimal individual benefit of the supervisor and postgraduate in three game scenarios.

The results in the three game scenarios are also illustrated in Figure 1, Figure 2 and Figure 3. It is worth mentioning that all the variables were dimensionless, but had relative sense.

Figure 1 depicts the optimal academic level of the community over time in the three game scenarios. As can be seen, the optimal academic level gradually rose until reaching the stable state. Moreover, the cooperative game quite obviously generated the highest optimal academic level, followed by the Stackelberg game, and finally the non-cooperative game. This finding demonstrated that, if the supervisor can work in a cooperative way with the postgraduate, or if the supervisor can share the cost with the postgraduate by, for example, improving the academic conditions and/or providing economic assistance, then the postgraduate’s potential can be released to some extent, and the academic level could be greatly improved.

Figure 2 shows the optimal total benefits of both the supervisor and the postgraduate in the three game scenarios. As can be seen, the cooperative game can obtain the maximum total benefit, while the non-cooperative game generates the minimum benefit. This finding demonstrates that the cooperative game can obviously enhance the benefits of both parties. Moreover, the result indicates that when the supervisor shares part of the cost with the postgraduate, or sacrifices their own benefit at first, the supervisor’s benefits will eventually be enhanced. In the non-cooperative game, when both actors only consider their own benefit, while disregarding the other’s benefit, the total benefit of the community will inevitably shrink. For example, if, for private benefit, the supervisor requires the postgraduate to do non-academic work or academic work that is not relative to the postgraduate’s topic, the postgraduate could be slack and inefficient in doing their work.

Figure 3 presents the optimal individual benefit of the supervisor and postgraduate in the three game scenarios. One can observe that in the non-cooperative and cooperative game scenarios, the supervisor’s benefits are always higher than those of the postgraduate. In the Stackelberg game scenario, as the supervisor shares a portion of the cost with the postgraduate in the initial stage, the supervisor’s benefit will be temporarily lower than that of the postgraduate. However, after the initial stage, the supervisor’s benefit grows rapidly, and exceeds that of the postgraduate when arriving at the stable stage. In the cooperative and Stackelberg game scenarios, the benefits of the supervisor and postgraduate both exceed those accrued in the non-cooperative mode. This finding implies that the supervisor creating a cooperative relationship and sharing the cost with the postgraduate could enhance the benefits of both sides, thus realizing a win–win situation.

## 6. Results with Different Parameters

This section discusses the influence on critical indicators—the optimal academic level and total benefit of the community in the three game scenarios—when the typical model parameters changed. These parameters include the cost coefficients of the positive and negative efforts of both actors ($c_{t,p}$, $c_{s,p}$, $c_{t,n}$, $c_{s,n}$), the benefit coefficients of both actors’ efforts ($k_{t,p}$, $k_{t,n}$, $k_{s,p}$, $k_{s,n}$), the decay factor of the academic level (δ), the effectiveness coefficients of the academic level change ($\lambda_{t}$, $\lambda_{s}$) and the supervisor’s sharing cost ratio in the Stackelberg game (i).

### 6.1. Results with Different Cost Coefficients of Efforts ($c_{t,p}$, $c_{s,p}$, $c_{t,n}$, $c_{s,n}$)

**Figure 4:**
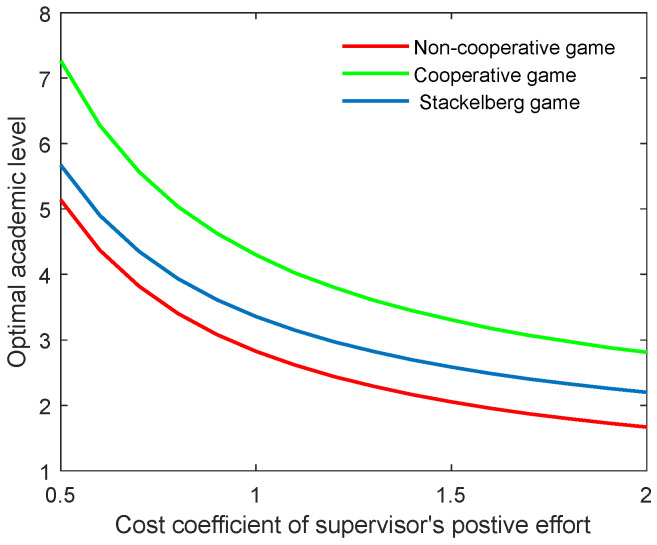
Optimal academic level with varying cost coefficients of supervisor’s positive effort $c_{t,p}$ for three game modes.

**Figure 5:**
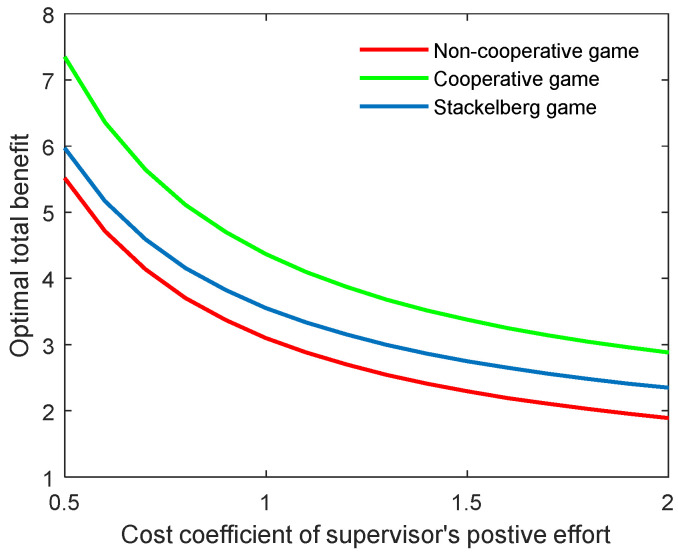
Optimal total benefit with varying cost coefficients of supervisor’s positive effort $c_{t,p}$ for three game modes.

As the supervisor’s benefit is related to the cost of the corresponding effort, this means that when the supervisor’s cost is increased, he or she will try to reduce the effort to improve the individual benefit. This could lead to a reduction in both the academic level and the community’s total benefit. Figure 4 and Figure 5 illustrate the optimal academic level and total benefit of the community with the varying coefficient of the supervisor’s positive effort, respectively. The results indicate that as the coefficient $c_{t,p}$ grows, the two indicators both reduce in all three game scenarios. This can also be explained by the state equation of the supervisor–postgraduate system (see Equation (3a)), and the benefit function (see Equation (4)). When the coefficient $c_{t,p}$ is improved, the supervisor’s cost will be increased, and thus, the integral value will diminish accordingly.

**Figure 6:**
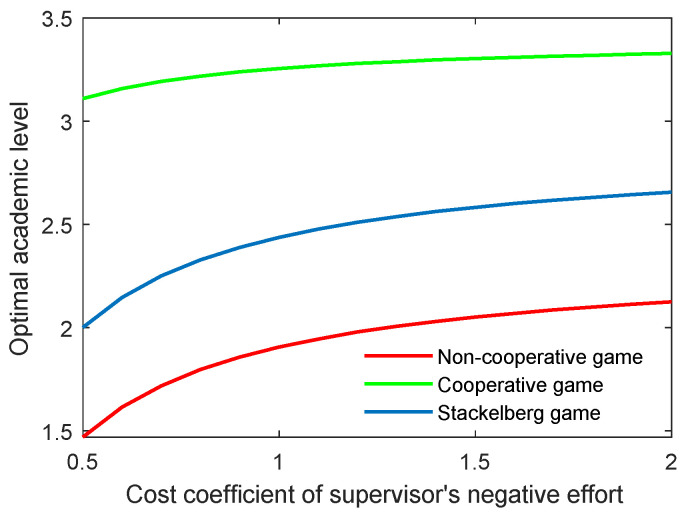
Optimal academic level with varying cost coefficients of supervisor’s negative effort $c_{t,n}$ in three game scenarios.

**Figure 7:**
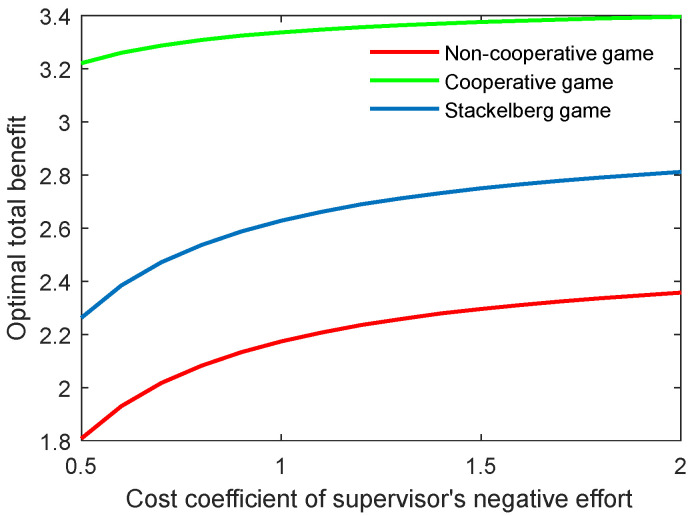
Optimal total benefit with varying cost coefficients of supervisor’s negative effort $c_{t,n}$ in three game scenarios.

The effect of the cost coefficient of the supervisor’s negative effort on the critical indicators is contrary to the effect of the supervisor’s positive effort. As shown in Figure 6 and Figure 7, when the coefficients ($c_{s,p}$, $c_{s,n}$) grew, the two indicators both increased for the three game modes. However, the growth rate of both the optimal academic level and the total benefit of the community gradually slowed in line with the increasing cost coefficient.

**Figure 8:**
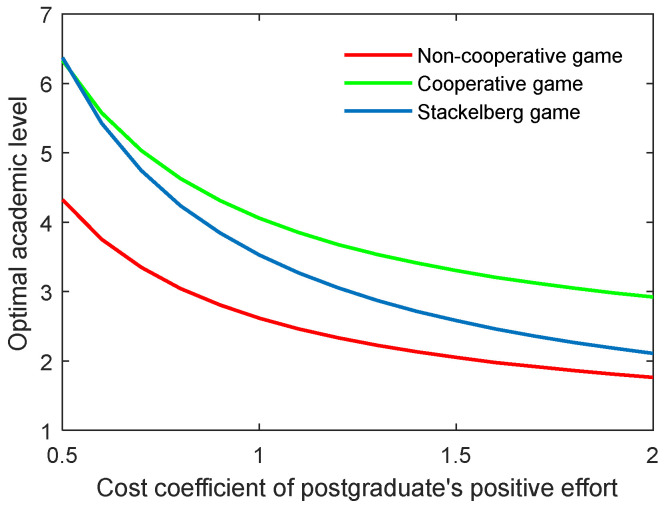
Optimal academic level with varying cost coefficients of postgraduate’s positive effort $c_{s,p}$ in three game scenarios.

**Figure 9:**
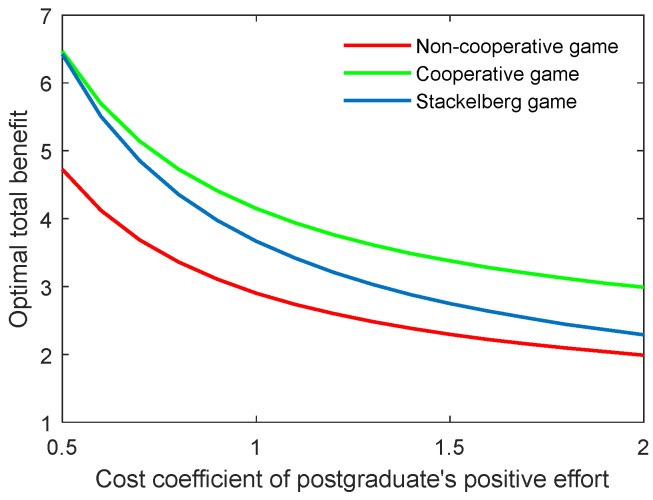
Optimal total benefit with varying cost coefficients of postgraduate’s positive effort $c_{s,p}$ in three game scenarios.

**Figure 10:**
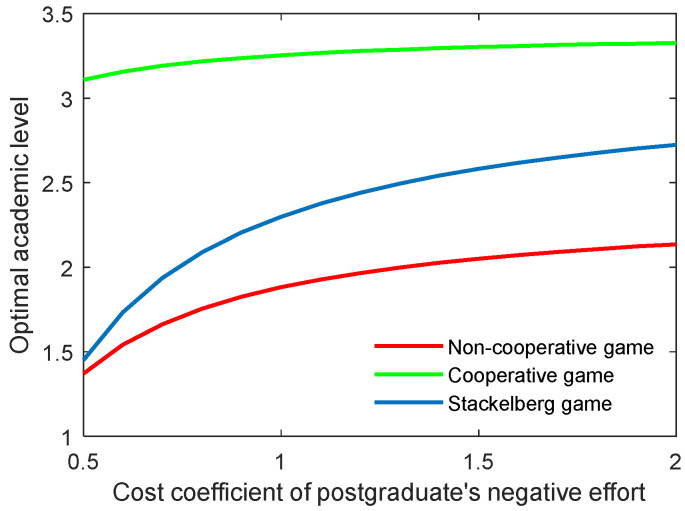
Optimal academic level with varying cost coefficients of postgraduate’s negative effort $c_{s,n}$ in three game scenarios.

**Figure 11:**
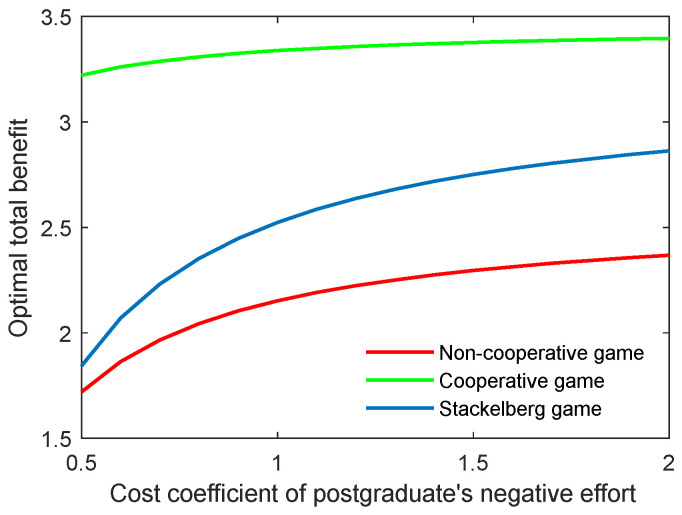
Optimal total benefit with varying cost coefficients of postgraduate’s negative effort $c_{s,n}$ in three game scenarios.

As for the cost coefficient of the postgraduate’s positive and negative efforts, similar conclusions can be generated as with those of the supervisor, as illustrated in Figure 8, Figure 9, Figure 10 and Figure 11. It is worth mentioning that for the Stackelberg game, the increasing and decreasing rate of the optimal academic level and total benefit of the community due to the postgraduate’s efforts were different from those in the non-cooperative and cooperative game. For the Stackelberg game, when the cost coefficient of the postgraduate’s positive effort increases, the effect of the supervisor’s sharing of the cost with the postgraduate will be additionally increased, apart from the previous terms in the objective function. This can create a new balance between the two actors and different growing and shrinking rates of the indicators.

### 6.2. Results with Different Benefit Coefficients of the Efforts ($k_{t,p}$, $k_{t,n}$, $k_{s,p}$, $k_{s,n}$)

**Figure 12:**
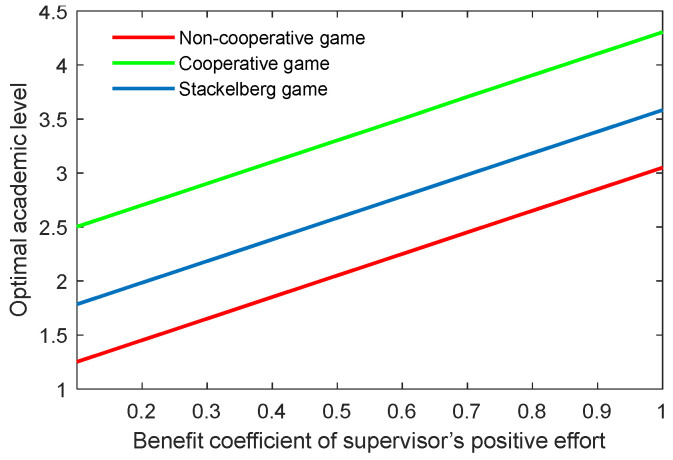
Optimal academic level with varying benefit coefficients of supervisor’s positive effort $k_{t,p}$ in three game scenarios.

**Figure 13:**
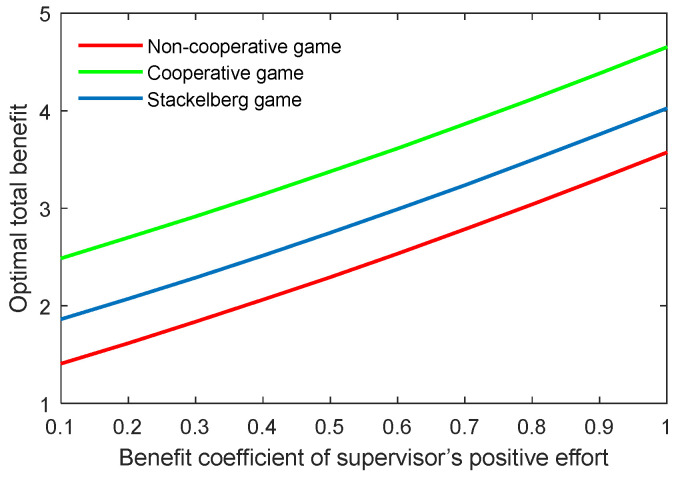
Optimal total benefit with varying benefit coefficients of supervisor’s positive effort $k_{t,p}$ in three game scenarios.

**Figure 14:**
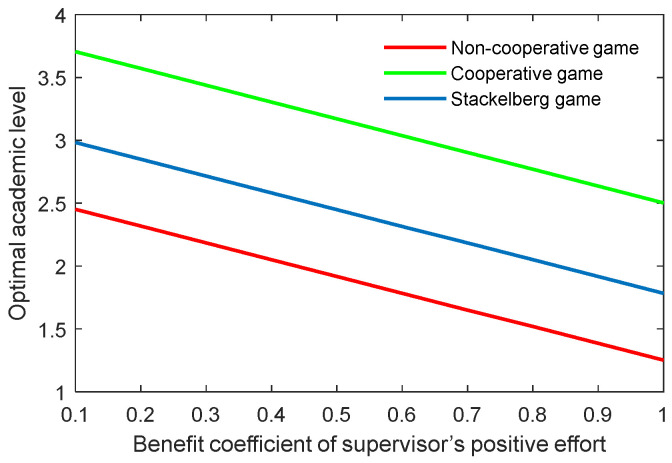
Optimal academic level with varying benefit coefficients of supervisor’s positive effort $k_{s,n}$ in three game scenarios.

**Figure 15:**
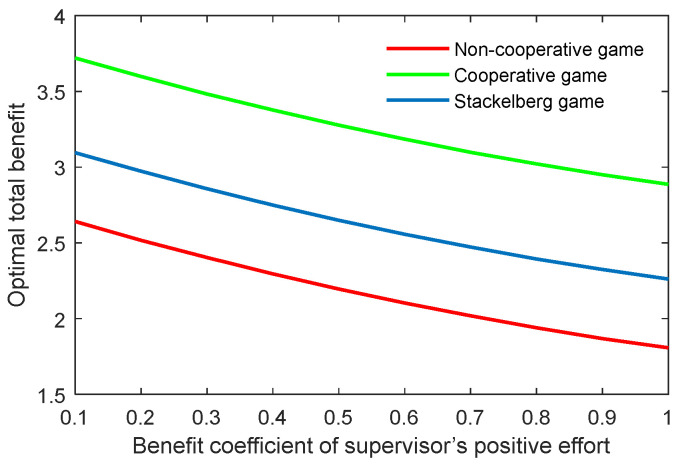
Optimal total benefit with varying benefit coefficients of supervisor’s negative effort $k_{s,n}$ in three game scenarios.

The benefit coefficient of the efforts is a reflection of the effectiveness and impact of the supervisor and postgraduate’s efforts on the benefit. The optimal academic level and total benefit with different benefit coefficients of the supervisor’s positive and negative efforts ($k_{t,p}$, $k_{t,n}$) are shown in Figure 12, Figure 13, Figure 14 and Figure 15. It was evident that two indicators almost linearly increased or declined in line with the growing or reducing benefit coefficients of efforts in the three game scenarios. This was because the change of the coefficients $k_{t,p}$ and $k_{t,n}$ can cause corresponding changes to the benefits due to the supervisor’s positive and negative efforts.

**Figure 16:**
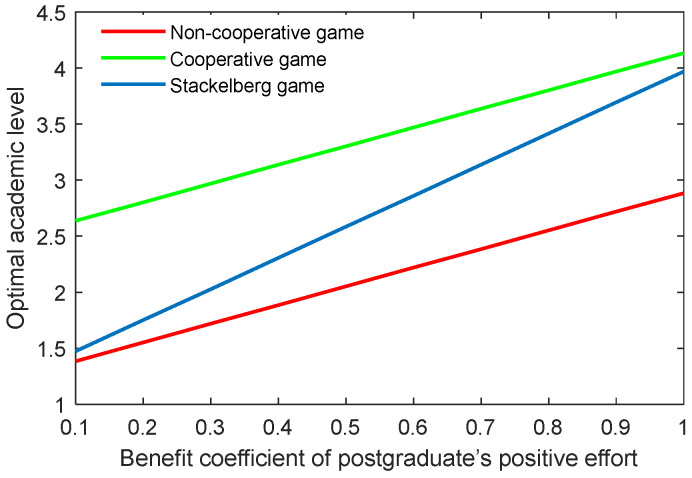
Optimal academic level with varying benefit coefficients of postgraduate’s positive effort $k_{s,p}$ in three game scenarios.

**Figure 17:**
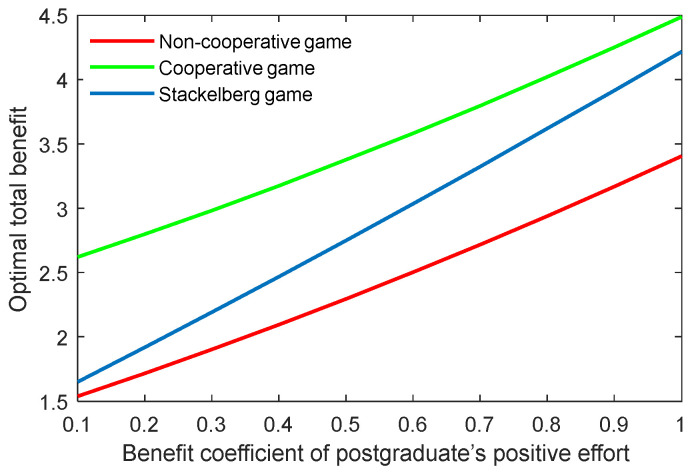
Optimal total benefit with varying benefit coefficients of postgraduate’s positive effort $k_{s,p}$ in three game scenarios.

**Figure 18:**
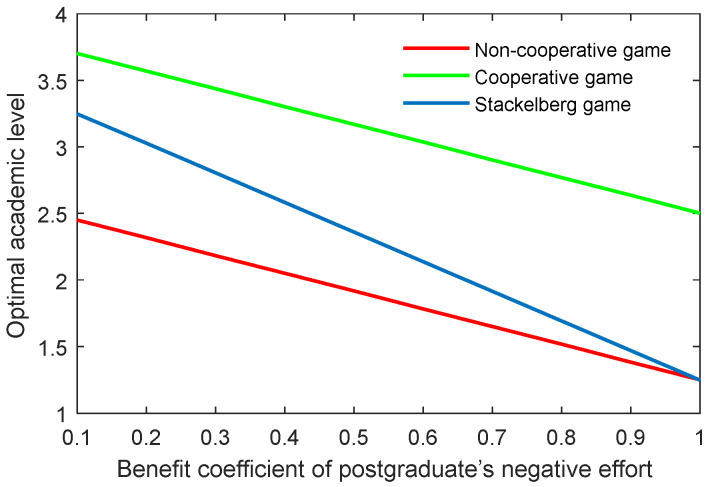
Optimal academic level with varying benefit coefficients of postgraduate’s negative effort $k_{s,n}$ in three game scenarios.

**Figure 19:**
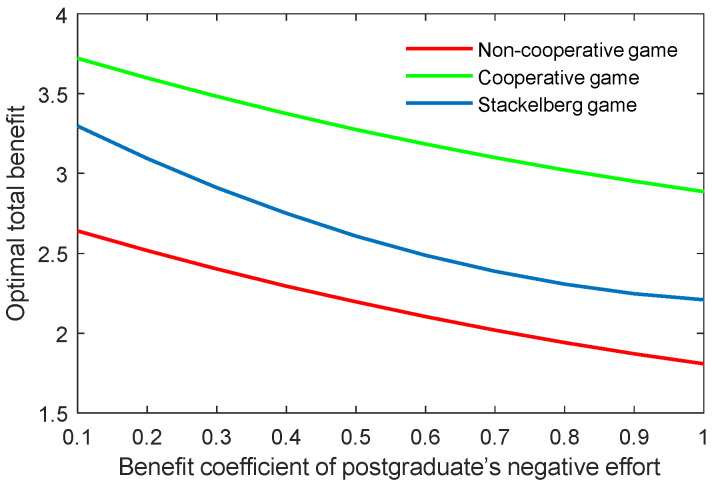
Optimal total benefit with varying benefit coefficients of postgraduate’s negative effort $k_{s,n}$ in three game scenarios.

The results of the benefit coefficient of the postgraduate’s positive and negative efforts are shown in Figure 16, Figure 17, Figure 18 and Figure 19. The results show that the non-cooperative and cooperative game scenarios generated similar effects to those of the supervisor’s positive and negative efforts. However, the exception was the case of the Stackelberg game scenario. On the one hand, the growth rate and shrinking rate of the optimal academic level and total benefit in the Stackelberg game scenarios were higher than those of the other two game scenarios for the benefit coefficient of the postgraduate’s positive and negative efforts, respectively.

### 6.3. Results with Different Decay Factors of the Academic Level (δ)

**Figure 20:**
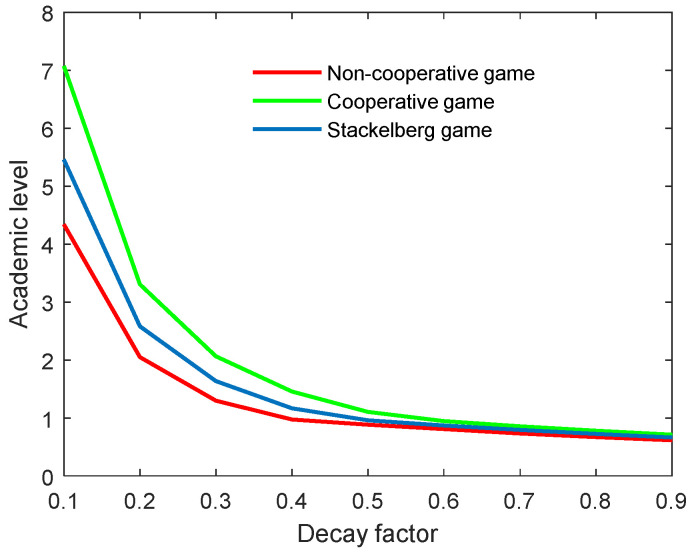
Optimal academic level with varying decay factors δ for three game modes.

**Figure 21:**
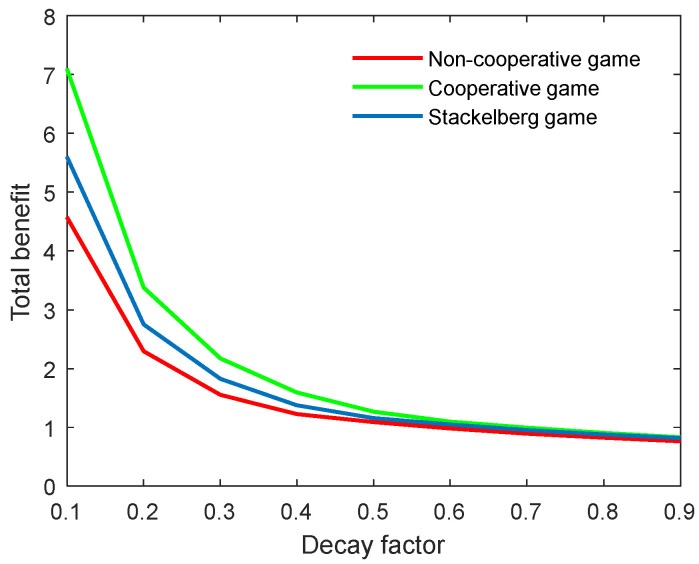
Optimal total benefit with varying decay factors δ for three game modes.

According to Figure 20 and Figure 21, both the optimal academic level and total benefit of the community diminish in line with the growing decay factor. Actually, this parameter is an index that reflects the natural decay of academic level over time. A high decay factor always means a rapid rate of decay in the research topic; for example, the weakening innovation and outdated academic work. Moreover, one can observe that the slope of both curves in the non-cooperative game was the biggest, followed by the Stackelberg mode, and finally the cooperative mode. This means that the optimal academic level and total benefit were more influenced in the non-cooperative game, and least influenced in the cooperative mode. However, when the decay factor was increased to a specific level, the factor’s influence on both indicators was almost unchanged for the three game modes.

### 6.4. Results with Different Efficiency Coefficients of the Academic Level Change ($\lambda_{t}$, $\lambda_{s}$)

**Figure 22:**
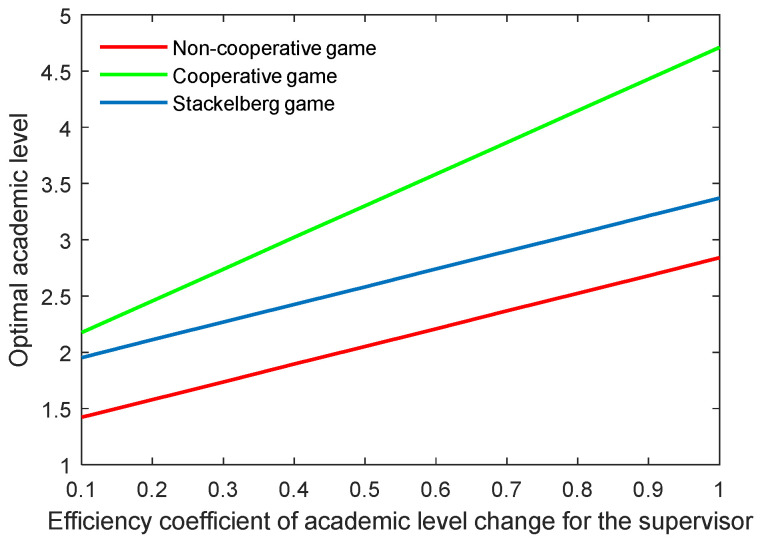
Optimal academic level with varying effectiveness coefficients of academic level change for the supervisor $\lambda_{t}$.

**Figure 23:**
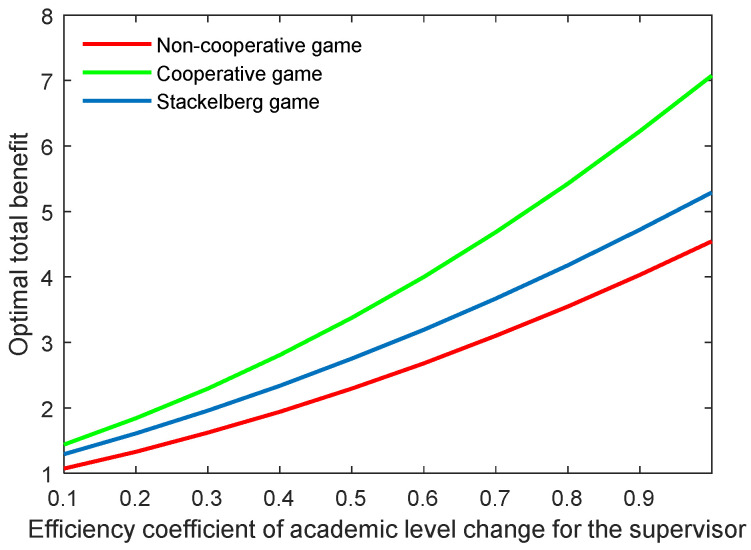
Optimal total benefit with varying effectiveness coefficients of academic level change for the supervisor $\lambda_{t}$.

**Figure 24:**
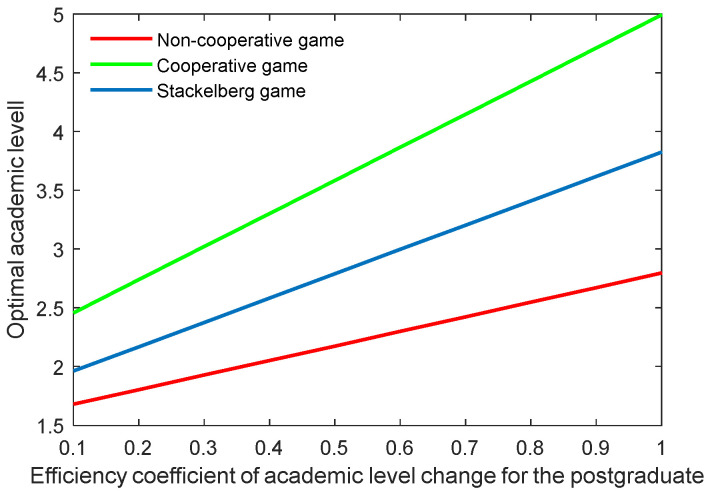
Optimal academic level with varying effectiveness coefficients of academic level change for the postgraduate $\lambda_{s}$.

**Figure 25:**
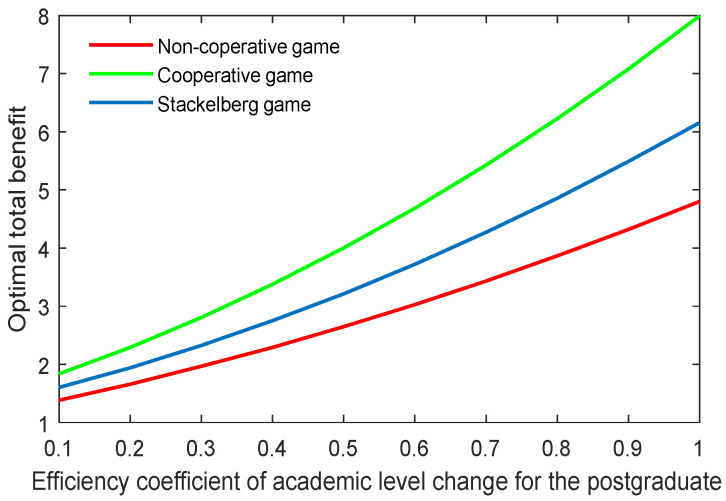
Optimal total benefit with varying efficiency coefficients of postgraduate’s academic level $\lambda_{s}$.

Figure 22, Figure 23, Figure 24 and Figure 25 show the optimal academic level and total benefit of the community, along with the varying effectiveness coefficient of the academic level change. This parameter reflects the effectiveness with regard to the benefit when the academic level improves or declines. It is noticeable that the optimal academic level and total benefit were positively correlated with this parameter. Among the three game scenarios, when improving the postgraduate’s effectiveness coefficient, the cooperative game will produce the highest improvement of the optimal academic level and total benefit of the community, followed by the Stackelberg game and, finally, the non-cooperative game.

### 6.5. Results with Different Cost Sharing Ratios i in the Stackelberg Scenario

**Figure 26:**
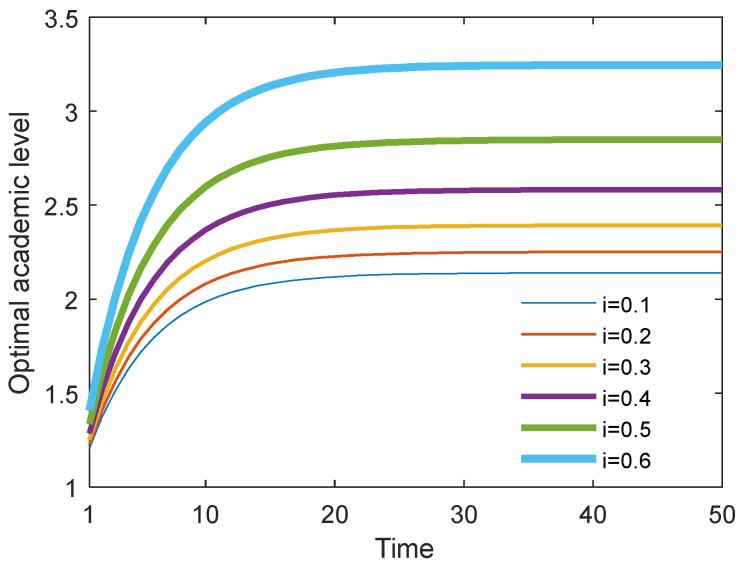
Optimal academic level with different cost sharing ratios.

**Figure 27:**
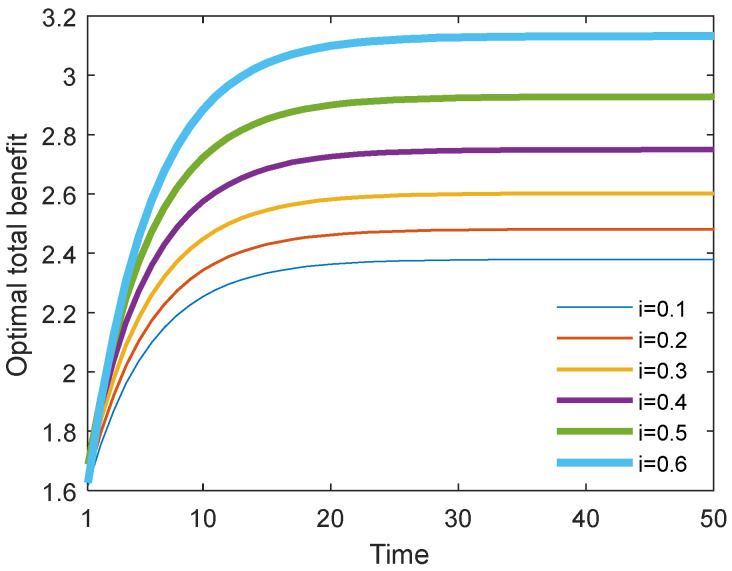
Optimal total benefits with different cost sharing ratios.

**Figure 28:**
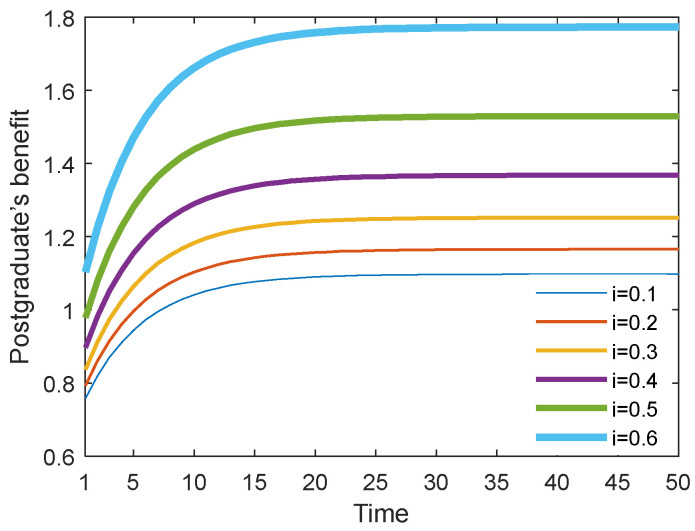
Postgraduate’s benefit with different cost sharing ratios.

**Figure 29:**
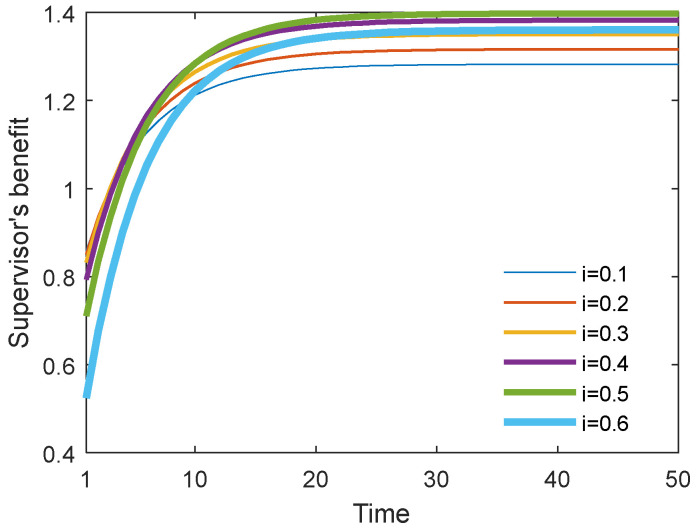
Supervisor’s benefit with different cost ratios.

Figure 26 and Figure 27 show the optimal academic level and total benefit of the community with the varying sharing cost ratio over time for the Stackelberg game. Figure 28 and Figure 29 show the individual benefit of the supervisor and postgraduate, respectively. The results indicate that as the sharing ratio grows, both the optimal academic level and total benefit are improved. This is due to the fact that if the supervisor shares part of the cost with the postgraduate, the postgraduate’s potential and creativity can be inspired, which can promote academic improvement. Moreover, one can observe that as the sharing ratio increases, the postgraduate’s benefit grows accordingly (see Figure 28). However, when this parameter was increased to 0.6, the supervisor’s benefit appeared to decline (see Figure 29). This is because the supervisor shared too much of the cost, and this lead to the reduction in the supervisor’s own benefit. This finding indicates that it is necessary for the supervisor to share the cost with the postgraduate to stimulate the postgraduate’s academic enthusiasm, but this ratio should be controlled within a reasonable range; otherwise, it will backfire and affect the supervisor’s benefit.

## 7. Conclusions

This paper established a differential model of the supervisor–postgraduate relationship, based on system dynamics. The mathematical model considers the supervisor’s and postgraduate’s positive and negative efforts as the control variables; the academic level is the state variable of the supervisor–postgraduate community. Then, a differential game was used to discuss the relationship in three scenarios, including non-cooperative, cooperative and Stackelberg game modes. The optimal equilibrium strategies are solved, respectively, and the results were analyzed. The main conclusions are summarized as follows:

1. A comparison of the results in the three game scenarios indicated that the cooperative game can achieve the highest optimal academic level and total community benefit, followed by the Stackelberg game and, finally, the non-cooperative game.
  (A) The quantitative results show that compared to the cooperative game, the non-cooperative game greatly curbs the positive efforts but enlarges the negative efforts of both actors. Specifically, in the non-cooperative game scenario, the supervisor’s positive effort was reduced by 22%, and the negative effort is increased to nine times that of the positive effort. Meanwhile, the postgraduate’s positive effort was reduced by 25%, and the negative effort grew to about ten times that of the positive effort. As a result, the optimal academic level and total benefit of the community in the non-cooperative game were reduced by 38% and 32%, respectively, when compared to those of the cooperative game.
  (B) For the supervisor-led Stackelberg game, when compared to the non-cooperative game, the supervisor’s positive and negative efforts remain unchanged, whereas the postgraduate’s positive and negative efforts were both increased by about 67%. Consequently, the optimal academic level of the community is improved by 26%, and the optimal total benefit of the community was improved by 20%. In short, the benefits of both the supervisor and the postgraduate were improved in the Stackelberg game.
  (C) Compared to the Stackelberg game, in the cooperative game, the supervisor’s positive effort was increased by 28%, while the negative effort was reduced by 67%. The postgraduate’s positive and negative efforts were decreased by 20% and 83%, respectively. This can lead to a 23% improvement of the optimal total benefit and a 28% improvement of the optimal total benefit of the community. Obviously, both the supervisor and postgraduate’s benefits were also enhanced in the Stackelberg game. This finding indicates that when the supervisor and postgraduate both cooperate, this was conductive to improving their respective benefits and the optimal total benefits of the community. In the cooperative scenario, the supervisor was more willing to actively mentor the postgraduate, and the postgraduate was also more willing to actively make progress on the research. Conversely, if both the supervisor and postgraduate go their own ways and ignore the other party, the optimal total benefit of the community will be at the lowest level. For example, if, for private profit, the supervisor compels the postgraduate to carry out non-academic work, then the postgraduate may passively cope with and even have a rebellious mentality towards the scientific research. This will waste much time and resources, and will ultimately weaken their respective benefits, as well as the total benefit of the community.
2. The growing rate of the optimal academic level and total benefit of the community in the three game scenarios all experience an increase first and then a decrease, before finally tending to a level stage. In the initial stage of the game, the optimal total benefit increased at a rapid pace via the supervisor’s and postgraduate’s respective efforts, for example, by working hard on the academic activity. However, the optimal total benefit only grew slowly over time.
3. For both the non-cooperative and cooperative game, the supervisor’s benefits were higher than those of the postgraduate. For the Stackelberg game, as the supervisor shared the cost with the postgraduate at the early stage, the supervisor’s benefit was temporarily less than that of the postgraduate. However, as time went on, the benefits of both actors grew, and the supervisor’s benefit eventually exceeded that of the postgraduate. Additionally, the results indicate that a proper improvement in terms of the supervisor’s sharing cost ratio will not only improve the postgraduate’s benefit but will also increase the supervisor’s benefit, thereby realizing a win–win condition.
4. The influences of different model parameters were also discussed. The correlation between the model parameters and the critical indicators, including the optimal academic level and the total benefit of the community, were presented. Particularly, for the Stackelberg game, when the sharing cost ratio was increased to a specific level, the supervisor’s benefit will not be further improved.

The above conclusions can have some practical implications. First, both the supervisor and postgraduate should avoid the events which damage the cooperative relationship. For example, the supervisor should not abuse their supervisory position by compelling the postgraduate to carry out scientific projects which can bring benefit for the supervisor but are not related to the postgraduate’s research topic. Instead, the supervisor should establish a proper stimulus mechanism, including material and spiritual incentives. Specifically, the supervisor can acquire or rent advanced experimental equipment in his or her own capacity and provide persistent and valuable guidance, as well as spiritual inspiration, to the postgraduates. Meanwhile, postgraduates should also actively communicate with the supervisor about the research progress and even about difficulties they are facing in life. Moreover, the university should try to promote an equal and free academic environment. For example, the university could invest more funds to improve the research conditions and made reasonable academic evaluation indicators to alleviate the research pressure on both the supervisors and postgraduates. This would help to create a free academic atmosphere and harmonious relationships.

## 8. Limitations and Future Research

In this study, the assumption was made that the supervisor and postgraduate were both rational when they made decisions. However, in fact, the decisions of the two parties were more or less influenced by their emotions at the time. The personalities of the supervisor and postgraduate, which have an impact on their relationship, were also neglected. Moreover, the supervisor–postgraduate relationship is influenced by many other factors, such as their individual cultural backgrounds. In a competitive environment, the supervisor and postgraduate will both face higher academic evaluation pressure, and so, their relationship may experience more disharmony than would be the case in a relatively relaxing circumstance. Therefore, future research will build a more precise supervisor-postgraduate game model that considers more factors, such as each individual’s cultural background and personality.

## Figures and Tables

**Table 1:** Results of optimal equilibrium strategies in the three game scenarios.

Indicator	Non-Cooperative Game	Cooperative Game	Stackelberg Game
Supervisor’s positive effort	0.5152	0.6606	0.5152
Supervisor’s negative effort	0.1455	0.0485	0.1455
Postgraduate’s positive effort	0.4545	0.6061	0.7576
Postgraduate’s negative effort	0.1697	0.0485	0.2828
Academic level of the community	2.0515	3.3029	2.5828
Supervisor’s sharing ratio of cost	-	-	0.4
Supervisor’s benefit	1.2518	1.7921	1.3823
Postgraduate’s benefit	1.0435	1.5839	1.3675
Total benefit of both	2.2953	3.3761	2.7498

## Data Availability

Not applicable.
